# Supplementary material for: Gag proteins encoded by endogenous retroviruses are required for zebrafish development
Source: bioRxiv. 2024 Mar 25:2024.03.25.586437. Preprint. [Version 1] doi: 10.1101/2024.03.25.586437 (PMC10996621; doi:10.1101/2024.03.25.586437)
Supplement: Supplement 2 [file media-2.pdf]

Supplementary Data Files

Supplementary Data 1.
Aligned fasta files and corresponding phylogenetic trees from A) Bik-1 and B) Bik-2 insertions in *Danio rerio* reference genome (GCF_000002035.6_GRCz11).

Supplementary Data 2.
BAM files mapping full-length proviral Bik-1 and Bik-2 insertions in the *Danio rerio* reference genome to various other *D. rerio* genome assemblies, providing evidence for presence of polymorphic loci.

Supplementary Data 3.
Differential enrichment analysis of RNA-seq data from Bik-1 LNA knock-down experiment with time point differences regressed out.

Supplementary Data 4.
A) Aligned fasta file and corresponding phylogenetic tree of ENS-3 and related retroelement reverse transcriptase domains. B) Aligned fasta file and phylogenetic tree of ENS-1 insertions in the jungle fowl genome (GCF_000002315.6).
